# Obstetric Use of Prostaglandin Preparations Compared to Mechanical Methods for Cervical Ripening in Pregnancies With Premature Rupture of Membranes at Term

**DOI:** 10.7759/cureus.84050

**Published:** 2025-05-13

**Authors:** Nobuko Yokoyama, Shunji Suzuki

**Affiliations:** 1 Obstetrics and Gynecology, Nippon Medical School, Tokyo, JPN; 2 Maternal and Child Health, Japan Association of Obstetricians and Gynecologists, Tokyo, JPN

**Keywords:** cervical ripening, dinoprostone, mechanical methods, premature rupture of the membranes, term

## Abstract

Background

We examined the difference in obstetric outcomes between the cases using dinoprostone and those using mechanical methods in pregnant women requiring cervical ripening following premature rupture of the membranes (PROMs) at term.

Methodology

During the study period, dinoprostone was used in 34 nulliparous women, while mechanical methods were used in 35 nulliparous women for cervical ripening following PROM at term. We examined the differences in the delivery outcomes between the two groups.

Results

On the day of induction start, 2 cases (6%) in the dinoprostone group were delivered by cesarean section due to non-reassuring fetal status (NRFS), while no cases were complicated by NRFS in the mechanical methods group (*P* = 0.15). However, there was no significant difference in the rate of cesarean delivery between the two groups (*P* = 0.73). In the mechanical methods groups, 3 cases (9%) were complicated by clinical intrauterine infection, while there was no case of clinical intrauterine infection in the dinoprostone group (*P* = 0.08). The clinical intrauterine infection in the cases of the mechanical methods group occurred more than 2 days after the start of cervical ripening; however, there was no case of neonatal infection in the two groups.

Conclusions

There were differences in the characteristics of the effects between the two methods in pregnant women with PROM who have an unfavorable cervix; however, there were no differences in the final perinatal outcome.

## Introduction

For pregnant women with an unfavorable cervix and labor that needs to be induced, cervical ripening before labor acceleration is usually the first step. In Japan, mechanical methods such as the insertion of laminaria tents or equivalent synthetic tents into the cervical canal have traditionally been used for cervical ripening [1,2]. However, since 2020, one formulation of dinoprostone, a controlled-release vaginal delivery system (PROPESS; Ferring Pharmaceuticals, Saint-Prex, Switzerland), has been available [1-8]. Researchers have reported that both options are similarly effective, and a combination of the two can be successful for cervical ripening in pregnant women with an unfavorable cervix without premature rupture of membranes (PROM) at term [9-11]. On the other hand, compared to cases using only pharmacologic agents (i.e., dinoprostone), the incidence of maternal and neonatal infection appears to be higher in those using mechanical methods, particularly in cases with PROM [12]. Some reports suggest that mechanical cervical ripening in pregnant women with premature rupture of membranes does not increase the risk of infection but also does not shorten the time to delivery [13], offering no clear benefit; however, no consensus has been reached. Because mechanical cervical dilation after membrane rupture has not generally been considered standard practice, comparisons limited to PROM cases have not been well investigated.

Based on this background, we examined differences in obstetric outcomes, including the incidence of intrauterine infections, between cases using dinoprostone and those using mechanical methods for cervical ripening in pregnancies with PROM at term.

## Materials and methods

This cohort study was approved by the Ethics Committee of the Japanese Red Cross Katsushika Maternity Hospital (K24-12). During the study period, subject assignment was based on the clinical policy in place at the time of admission. All pregnant women at our institution provided prior informed consent for the retrospective review of their obstetric outcomes.

The study was conducted at our institute, which is one of the main perinatal centers in Tokyo, Japan, between January 2023 and July 2024. Clinical data on maternal characteristics and obstetric outcomes were obtained from the hospital records. At our institution, in cases of PROM beyond 37 weeks of gestation, labor is induced at 9:00 a.m. on the day following PROM if there are no signs of labor onset or indications of intrauterine infection, such as maternal fever of 38 °C or higher or tachycardia in either the mother or fetus [12-16]. Therefore, we excluded the cases in which labor had started before the next day and/or the cases in which intrauterine infection was suspected. In addition, the cases with a Bishop score of 7 or higher at the start of induction were excluded from the study. The cases with perinatal complications such as hypertensive disorders and oligohydramnios were also excluded. In addition, cases with a Bishop score of 0-1, in which little cervical ripening was observed, were excluded from this study. This is because, for such cases, our institute had only performed mechanical methods for cervical ripening based on some previous reports [17,18]. Therefore, the criteria for inclusion in this study were as follows: nulliparous singleton pregnancy, cephalic presentation, gestational age of 37-41 weeks, PROM, and a Bishop score between 2 and 6 at the beginning of induction. During the study period, intravenous antimicrobials were initiated on the morning following PROM.

During the study period, 34 nulliparous women met this criterion and received dinoprostone for cervical ripening following PROM, while mechanical methods were used in 35 women (i.e., insertion of laminaria tents or equivalent synthetic tents such as Dilapan into the cervical canal) [6]. To insert the PROPESS, it was held between the second and third fingers of a gloved hand and placed in the posterior fornix of the vagina. If 12 hours had elapsed after insertion, the PROPESS was removed regardless of whether cervical ripening had occurred. The criteria for removal of PROPESS included: (1) uterine contractions every 3 minutes for 30 minutes, (2) tachysystole, (3) non-reassuring fetal status, and (4) appearance of indefinite maternal complaints. On the other hand, before the insertion of Dilapan, the vaginal canal was washed with saline using a vaginal speculum. Then, the cervix was held with a single-tooth forceps, and each Dilapan rod was grasped with forceps and slowly inserted into the cervix, for a total of one to five insertions. The Dilapans were held in place with gauze moistened with saline solution. If these procedures did not result in effective labor, oxytocin was used for labor augmentation. In addition, if oxytocin had a poor effect, it was administered again the following day, provided there were no signs of infection.

We examined maternal age, parity, gestational age at PROM, maternal body mass index, and Bishop score at the start of induction; oxytocin use; mode of delivery (vaginal or cesarean); number of days from the start of induction to delivery; neonatal birth weight; neonatal asphyxia (defined as an Apgar score at 1 minute < 7); umbilical artery pH; and the incidence of two types of intrauterine infection. The first type was defined by clinical manifestations such as maternal fever of 38°C or higher with fetal tachycardia, and the second by signs of neonatal infection, such as respiratory distress [12-16]. For this study, the former was defined as clinical intrauterine infection, and the latter as fetal intrauterine infection.

Data were presented as mean ± standard deviation (SD) or numbers (percentages). Statistical analyses were performed with SAS version 8.02 (SAS Institute, Cary, NC). Cases were compared by unpaired t-test for continuous variables or the chi-square test for categorical variables. Statistical significance was set as *P* < 0.05.

## Results

Table 1 shows the clinical characteristics of the women in the dinoprostone and mechanical methods groups. There were no significant differences in these variables between the two groups.

**Table 1:** Clinical characteristics of the nulliparous women in the dinoprostone and mechanical methods groups. Data are presented as mean ± standard deviation (SD). *By unpaired *t*-test (*P*-values of <0.05 were considered significant).

	Dinoprostone	Mechanical methods	*t*-value*	*P*-value*
Total number	34	35		
Maternal age (years)	33.6 ± 7	32.8 ± 7	1.26	0.41
Gestational age at PROM (weeks)	39.3 ± 2	39.5 ± 2	-0.59	0.18
Body mass index at the start of induction	24.9 ± 3.5	25.2 ± 3.5	-0.67	0.48
Bishop score at the start of induction	2.0 ± 2	1.8 ± 2	0.59	0.40
Neonatal birth weight (weeks)	3143 ± 458	3169 ± 472	-5.01	0.11

Table 2 shows the obstetric outcomes of the delivery in the dinoprostone and mechanical methods groups. On the day of induction start, 2 cases (6%) in the dinoprostone group were delivered by cesarean section due to non-reassuring fetal status (NRFS), while no cases in the mechanical methods were complicated by NRFS on the day (*P* = 0.15). The rate of oxytocin use in the dinoprostone group was significantly lower than that in the mechanical methods group (38% vs. 65%, *P* = 0.02). There was no significant difference in the rate of cesarean delivery between the two groups (*P* = 0.73). In the cases of vaginal delivery, there was no significant difference in the number of days from the start of induction to delivery between the two groups (*P* = 0.36), while in the cases of cesarean delivery, the number of days from the start of induction to delivery in the dinoprostone group was significantly fewer than that in the mechanical methods groups (1.4 ± 1 vs. 2.5 ± 1, *P* = 0.03). In the mechanical methods groups, 3 cases (9%) were complicated by clinical intrauterine infection, while there were no cases of clinical intrauterine infection in the dinoprostone group (*P* = 0.08). The clinical intrauterine infection in the cases of the mechanical methods groups occurred more than two days after the start of cervical ripening. Finally, there was no case of neonatal infection in the both groups.

**Table 2:** Obstetric outcomes of the delivery in the dinoprostone and mechanical methods groups Data are presented as numbers (percentages) or mean ± standard deviation (SD). *Chi-square test for categorical variables continuous/unpaired *t*-test for continuous variables (*P*-values of < 0.05 were considered significant). NRFS, non-reassuring fetal status

	Dinoprostone	Mechanical methods	Chi-square value/*t*-value*	*P*-value*
Total number	34	35		
Cesarean delivery due to NRFS on the day of induction starts	2 (6)	0 (0)	2.12	0.15
Oxytocin use	13 (38)	23 (65)	5.22	0.02
Total vaginal delivery	22 (65)	24 (69)	0.12	0.73
Number of days from the start of induction to vaginal delivery (days)	1.2 ± 1	1.5 ± 1	-1.25	0.36
Total cesarean delivery	12 (35)	11 (31)	0.12	0.73
Number of days from the start of induction to cesarean delivery (days)	1.4 ± 1	2.5 ± 1	-4.57	0.03
Neonatal asphyxia	0 (0)	1 (3)	0.99	0.32
Umbilical artery pH < 7.1	1 (3)	0 (0)	1.04	0.31
Clinical intrauterine infection	0 (0)	3 (9)	3.05	0.08
Neonatal intrauterine infection	0 (0)	0 (0)	-	1

## Discussion

Based on the results of this study, the final obstetric outcomes showed no difference between the dinoprostone and mechanical methods groups in cases of PROM at term. However, although the difference was not significant, two cases in the dinoprostone group resulted in cesarean delivery due to NRFS associated with the uterine contractions at a time of insufficient cervical ripening at the day of dinoprostone use (day 1), while in the mechanical methods group, there were three cases showing signs of clinical intrauterine infection two or more days after the start of cervical ripening. Although the results could have been predicted from previous reports [9-12], the difference seemed to be associated with the difference in the number of days required for delivery in cases of cesarean section between the two groups.

To date, both options have been observed to be equally effective in a variety of pregnant women requiring cervical ripening for labor at term [10,11,19-21]. In addition, the use of vaginal dinoprostone in pregnant women undergoing induction of labor with PROM has been observed to be safe for both the mother and fetus, shorten total delivery time, and not increase the risk of cesarean section compared to women without PROM [7,22]. The present results support these previous observations [7,22].

The results of this study showed no significant difference in the final rate of vaginal delivery and the time required for vaginal delivery between the two groups following PROM at term. However, the course of labor appeared to have different characteristics, although the differences were not significant. For example, dinoprostone could have induced NRFS, although not as frequently, by excessive labor pains without sufficient cervical ripening. Such risks were also feared in a previous large study in cases requiring cervical ripening without PROM [23]. Although dinoprostone may be one of the safest and most effective methods to relieve pain during delivery, it has side effects such as transient uterine tachysystole and hyperstimulation [23,24]. It is undeniable that these may cause a different kind of stress to the fetus than intrauterine infections observed in the cases of mechanical methods. In addition, while these complications may be unavoidable, it is anticipated that adverse perinatal outcomes can be avoided with continuous fetal heart rate monitoring. Although both patients and physicians should be aware of the associated risks, dinoprostone may be a useful option for cervical ripening in cases of PROM at term. In addition, dinoprostone has the advantage over mechanical methods in that it may not impose pain stress on pregnant women during use [25].

On the other hand, the incidence of clinical symptoms suggesting intrauterine infection seemed to be higher in patients who underwent mechanical methods than in those who used dinoprostone, especially in cases without delivery within two days after the start of cervical ripening. In an earlier study by Heinemann et al. [12], maternal and neonatal infectious morbidity appeared to be increased when mechanical agents were used for cervical ripening compared with the use of pharmacologic agents alone. In this study, there were no cases of fetal infection in either group; however, the current results may support the findings of the previous study [12]. In addition, the infrequent use of oxytocin in this study may have been an advantage of pharmacologic agents.

We acknowledge that the small sample size is a significant limitation of this study. With a larger number of subjects, a significant difference between the two groups might have been observed. However, we have suspended the use of these two ripening methods in a randomized trial, as it has become clear that each induction method has distinct characteristics. In clinical practice, informed consent for each method may be necessary, taking into account the unique features of each approach. We recognize that this is undeniably a potential confounding factor moving forward. However, for example, we prioritized the implementation of innovations that would favor mechanical methods in cases of oligohydramnios prone to NRFS and/or severely unfavorable cervix, where avoiding uterine contractions before cervical ripening is important. Additionally, mechanical methods were preferred in patients with conditions that would make the use of dinoprostone hesitant, such as a history of asthma.

Although the current study is small, the results may suggest different recommendations for the use of dinoprostone or mechanical methods in pregnant women with PROM and an unfavorable cervix. However, a larger prospective study is needed to provide clear clinical guidelines, such as recommending dinoprostone when faster ripening is desired.

## Conclusions

We examined the difference in obstetric outcomes between the groups using dinoprostone and those using mechanical methods for cervical ripening following PROM at term. Although there were no differences in the final perinatal outcomes between dinoprostone and mechanical methods in pregnant women with PROM and an unfavorable cervix, there were differences in the clinical characteristics of the effects.
